# Multi-institutional comparison of volumetric modulated arc therapy vs. intensity-modulated radiation therapy for head-and-neck cancer: a planning study

**DOI:** 10.1186/1748-717X-8-26

**Published:** 2013-01-31

**Authors:** Andrea Holt, Dirk Van Gestel, Mark P Arends, Erik W Korevaar, Danny Schuring, Martina C Kunze-Busch, Rob JW Louwe, Corine van Vliet-Vroegindeweij

**Affiliations:** 1 Department of Radiation Oncology, The Netherlands Cancer Institute – Antoni van Leeuwenhoek Hospital, Plesmanlaan 121, Amsterdam, CX 1066, The Netherlands; 2 Department of Radiation Oncology, Antwerp University Radiotherapy, UZA/ZNA, Antwerp, Belgium; 3 Radiotherapeutic Institute Friesland, Leeuwarden, The Netherlands; 4 Department of Radiation Oncology,University Medical Center Groningen, University of Groningen, Groningen, The Netherlands; 5 Department of Radiation Oncology, Catharina Hospital, Eindhoven, The Netherlands; 6 Department of Radiation Oncology, Radboud University Medical Center Nijmegen, Nijmegen, The Netherlands; 7 Present address: Department of Radiation Oncology, University of Duisburg-Essen, Essen, Germany; 8 Present address: Department of Radiation Oncology, Wellington Blood and Cancer Centre, Wellington, New Zealand

**Keywords:** Head-and-neck cancer, Simultaneous integrated boost, Volumetric modulated arc therapy, Intensity-modulated radiation therapy, Multi-institutional collaboration, SmartArc

## Abstract

**Background:**

Compared to static beam Intensity-Modulated Radiation Therapy (IMRT), the main advantage of Volumetric Modulated Arc Therapy (VMAT) is a shortened delivery time, which leads to improved patient comfort and possibly smaller intra-fraction movements. This study aims at a treatment planner-independent comparison of radiotherapy treatment planning of IMRT and VMAT for head-and-neck cancer performed by several institutes and based on the same CT- and contouring data.

**Methods:**

Five institutes generated IMRT and VMAT plans for five oropharyngeal cancer patients using either Pinnacle^3^ or Oncentra Masterplan to be delivered on Elekta linear accelerators.

**Results:**

Comparison of VMAT and IMRT plans within the same patient and institute showed significantly better sparing for almost all OARs with VMAT. The average mean dose to the parotid glands and oral cavity was reduced from 27.2 Gy and 39.4 Gy for IMRT to 25.0 Gy and 36.7 Gy for VMAT, respectively. The dose conformity at 95% of the prescribed dose for PTV_boost_ and PTV_total_ was 1.45 and 1.62 for IMRT and 1.37 and 1.50 for VMAT, respectively. The average effective delivery time was reduced from 13:15 min for IMRT to 5:54 min for VMAT.

**Conclusions:**

Independently of institution-specific optimization strategies, the quality of the VMAT plans including double arcs was superior to step-and-shoot IMRT plans including 5–9 beam ports, while the effective treatment delivery time was shortened by ~50% with VMAT.

## Background

A notable difficulty with irradiation of head-and-neck cancer (HNC) is the large number of organs at risk (OARs) in close proximity to regions with disease, including the salivary glands, spinal cord and brainstem, larynx and pharyngeal constrictors, oral mucosae, tongue and lips, masseter as well as eyes and inner ears. The challenging task for the treatment planner is to find the most optimal trade-off in sparing the different OARs for each individual patient. Often better sparing of one OAR implies sacrificing another OAR, and in most patients high-grade radiation-induced toxicity is unavoidable while ensuring sufficient dose coverage of the planning target volume (PTV). This may result in severe consequences for the quality of life of these patients.

Introduction of intensity-modulated radiation therapy (IMRT) techniques for the treatment of HNC replaced conventional 3D-conformal radiation therapy (3D-CRT) techniques, which resulted in much better dose conformity and sparing of the OARs and, therefore, less radiation-induced toxicity [1-3]. When using IMRT for irradiation of oropharyngeal cancer salivary function was less impaired, but the majority of the patients still suffered from some degree of xerostomia [4-6]. Braam *et al.* showed that the normal tissue complication probability (NTCP) at several time points after radiation therapy was less than 20% only if the mean dose to the parotid glands was lower than 25 Gy [7], a dose level that even with IMRT is often not achieved.

Recently, the next generation of IMRT techniques, volumetric modulated arc therapy (VMAT) has become widely available. Compared to static-beam IMRT, rotational VMAT is supposed to decrease treatment delivery times with at least similar or even better plan quality [8]. A number of single-institution studies have been published for VMAT for HNC [9-15]. The authors of these studies observed comparable or better PTV coverage and conformity as well as better sparing of OARs for VMAT compared to IMRT, while delivery times were shortened by 35-60%. Recently, a multi-institutional study comparing different treatment technologies planned in different institutes has been reported [16]. However, no multi-institutional studies comparing VMAT vs. IMRT planned by multiple institutes using the same set of patients and treatment planning engines have been reported so far.

The here presented study is the result of a collaboration of five institutes in the Netherlands with similar equipment. This collaboration aimed at a fast and safe clinical introduction of VMAT for HNC (implemented under the name SmartArc and Oncentra VMAT in the treatment planning systems Pinnacle and Oncentra Masterplan, respectively) on Elekta equipment and a high quality of VMAT treatment planning in all participating institutes. To evaluate the potential of VMAT all five participating institutes generated IMRT and VMAT plans based on their locally developed IMRT planning knowledge. The data retrieved from this multi-institutional collaboration gives the possibility of a treatment planner-independent comparison of VMAT vs. step-and-shoot IMRT for HNC. The reason for this is that each institute uses different optimization strategies and sets of objectives, and has different preferences for sparing OARs while identical patient data was used for treatment planning.

## Methods

### Patient selection and contouring

For this retrospective treatment planning study, computed tomography (CT) data including contouring of five patients with oropharyngeal cancer were selected (patient characteristics listed in Table 1). These patients were previously treated with the standard clinical protocols. According to the guidelines of the participating institutes, the patient data was properly anonimized and no informed consent of the patient was required. The planning target volumes (PTVs) and organs at risk (OARs), amongst others parotid glands, submandibular glands, oral cavity, larynx, pharyngeal constrictors and mandible, and if applicable, their corresponding substructures, were delineated by an experienced clinician. Delineation of an OAR was skipped in cases where the PTV completely encompassed this (sub-) structure.

**Table 1:** Patient characteristics

**Patient**	**Location**	**Classification**	**Lymph nodes involved**	**PTV**_**elective**_	**PTV**_**boost**_
				**volume (cm**^**3**^**)**	**volume (cm**^**3**^**)**
1	Base of tongue R	T1N2aM0	2 & 3 R	501	105
2	Tonsil R	T2N2cM0	3 R & 2 bilat	621	234
3	Tonsil L	T3N2cM0	1,2,3,4 bilat	992	381
4	Base of tongue R	T3N2cM0	1b R & 2 bilat	836	422
5	Tonsil L	T2N1M0	2 L	571	146
Average				704	258

### Dose prescription and plan acceptance parameters

Both VMAT and IMRT plans were generated for a treatment in 32 fractions, to deliver a total dose of 56 Gy to the PTV_elective_ (i.e. a fraction dose of 1.75 Gy) and a simultaneous integrated boost (SIB) to 69.12 Gy to regions with macroscopic disease (i.e. a fraction dose of 2.16 Gy). Primary goal of treatment planning was to cover at least 99% of the volume of PTV_elective_ and PTV_boost_ with 95% of the prescribed dose (53.2 Gy and 65.66 Gy, respectively), and to restrict the volume receiving more than 107% in the PTV_boost_ of the prescribed dose (73.96 Gy) to a total volume below 2 cm^3^ (all parameters summarized in Table 2). The maximal allowed point dose to OARs was 54 Gy for the brainstem and 50 Gy for the spinal cord. In addition, the mean dose preferably should be below 25 Gy for the parotid glands (for at least one parotid), submandibular glands and the oral cavity (if possible), and below 45 Gy for the larynx and pharyngeal constrictors.

**Table 2:** Treatment planning objectives

**Structure**	**Parameter**	**Objective**	**Priority**
PTV_boost_	Prescribed dose	69.12 Gy (32 * 2.16 Gy)	
	V_99%_	> 95% of prescribed dose (= 65.66 Gy)	High
	D_107%_	< 2 cm^3^	High
PTV_elective_	Prescribed dose	56 Gy (32 * 1.75 Gy)	
	V_99%_	> 95% of prescribed dose (= 53.2 Gy)	High
Spinal Cord	D_max_	< 50 Gy	High
Brainstem	D_max_	< 54 Gy	High
Parotid glands	D_mean_	Preferably < 25 Gy	High-Medium
		For at least the contralateral gland	
Submandibular glands	D_mean_	Preferably < 25 Gy	Medium
Oral cavity	D_mean_	Preferably < 25 Gy	Medium
Larynx	D_mean_	Preferably < 45 Gy	Medium-Low
Pharyngeal constrictors	D_mean_	Preferably < 45 Gy	Medium-Low
Other OARs			Low

### Treatment planning

The CT data sets of five patients including contouring were shared between the participating institutes (*n* = *5*). All institutes used their locally developed treatment planning technique and inverse planning objectives previously used with IMRT as starting point. VMAT and IMRT plans were generated using Pinnacle^3^ v9.0 (Philips Healthcare, Best, The Netherlands) (*n* = *4*) or alternatively Oncentra Masterplan (Nucletron, Veenendaal, The Netherlands) (*n* = *1*, institute C), which were commissioned for treatment delivery using Elekta linear accelerators (Elekta Oncology, Crawley, United Kingdom) equipped with either standard MLCs with 1 cm leaves not allowing interdigitation (*n* = *4*) or a beam modulator with 0.4 cm leaves allowing interdigitation (*n* = *1*, institute D).

Both treatment planning systems (TPSs) used in this study employ nearly identical optimization modules developed by RaySearch Laboratories (RaySearch Laboratories, Stockholm, Sweden) for VMAT planning: p-RayArc marketed as SmartArc in Pinnacle^3^, and n-RayArc marketed as Oncentra VMAT in Oncentra Masterplan; and for IMRT planning: p-RayMachine marketed as Direct Machine Parameter Optimization (DMPO) in Pinnacle^3^, and n-RayMachine marketed as Direct Step and Shoot (DSS) in Oncentra Masterplan.

With VMAT and IMRT treatment planning, each optimization is started with generation of fluence maps. After typically 10–15 iterations, at the so-called conversion iteration, segments are produced. Following an intermediate collapsed-cone dose calculation, optimization is continued using Direct Aperture Optimization (i.e., DMPO in Pinnacle^3^, and DSS in Oncentra Masterplan) up to a total of 30–50 iterations.

### IMRT planning parameters

Step-and-shoot IMRT plans were generated according to each institute’s practice using typically 5–9 coplanar beams (one institute used one additional non-coplanar beam at 330° at a couch angle of 90°) with a total of 50–70 segments. The lower limit for the segment size ranged from 4 to 9 cm^2^, with a minimum of 2 to 5 monitor units (MUs) per segment.

### VMAT planning parameters

VMAT plans were generated using one dual arc (i.e. a double arc generated from a single arc at the segmentation step) with an arc length close to 360°. Before starting the study, all institutes had individually tested different arc setups with, for example, only a single arc or one or more dual arcs with varying arc lengths or combinations thereof. During discussion of the first results within the workgroup it was concluded that the use of a dual arc over the full range would yield the most promising, clinically acceptable results. The final resolution of control points within the arc was set to 4°, which was found to allow sufficient modulation at still acceptable duration of the optimization. The collimator angle was typically set to a value between 10° and 30° (or alternatively between 330° and 350°) to avoid tongue-and-groove effects. All IMRT and VMAT plans were generated using 6 MV photons.

VMAT plan optimization was restarted typically three to five times after the initial run without prior resetting of the earlier optimization result including segments and dose rate along the arcs (so-called “warm re-starts”). At the end of each of these optimization steps a full collapsed-cone dose calculation is performed. After a restart of the optimization altered dose distributions after adaptation of segments are calculated as a small perturbation on the previously calculated dose distribution using a simple pencil-beam based approach. This approximation leads to an accumulation of errors in the dose distribution during the optimization process, which is then “repaired” by the forced intermediate dose calculation after a fixed number of iterations. This procedure greatly improves accordance with the planning objectives in each step since dose distribution after full dose calculation including inhomogeneity corrections will be close to the altered dose distribution found during the optimization process.

If necessary and desirable, target doses and/or objective weights were adapted during the optimization process and in-between re-starts to further lower dose to OARs and/or improve PTV coverage. However, in case of large changes in the objectives the optimization was repeated including the segmentation step, i.e. re-started from scratch.

### Planning objectives

Each institute started with their locally developed planning strategy including their specific set of objectives. After initial planning of one patient the planning procedures and parameters were discussed in the group and as a result, some institutes adjusted their individual planning parameters and set of objectives. Hereafter, each institute generated IMRT and VMAT plans for each of the five patients (including the initially planned patient if necessary) using a similar set of objectives for both IMRT and VMAT, with only small or no changes to objective doses and weights within the same patient.

A typical set of objectives to create a SIB plan always included for both PTV_elective_ and PTV_boost_ minimum and maximum dose objectives with high objective weights, i.e. 50–100 (if 100 would be the maximum for objective weights). In a limited number of institutes and cases, also uniform dose objectives were added. Furthermore, all institutes included maximum point dose objectives for spinal cord and brain stem with mostly intermediate objective weights, i.e. 5–50. Some institutes expanded these structures by 3–5 mm to create an extra safety margin, the so-called planning risk volume (PRV).

Dose to the other OARs including the left and right parotid, submandibular glands if delineated, oral cavity, larynx, pharyngeal constrictors and mandible was typically steered using maximum EUD objectives with mostly low objective weights, i.e. 1–10, and a gEUD factor of a = 1 (representing the mean dose). For each parotid, typically for the part of the parotid outside the PTV (or the PTV expanded by several mm) at least one dose volume histogram (DVH) point objective was added with mostly low objective weights, i.e. 1–10. In addition, all institutes used ring structures to create steep dose gradients outside the PTV_elective_ and also between the two dose levels. Here, mostly maximum point dose objectives with low to intermediate objective weights, i.e. 1–20 were used. In addition, some of the participating institutes used dose objectives to restrict dose to one or more of the following OARs: lung tops, shoulders, eyes and inner ears.

### Determination of effective delivery times

Delivery times for IMRT and VMAT plans were determined on Elekta linear accelerators without making use of the AFS (auto-field-setup) function to prepare beams and segments for delivery. Tests with enabled AFS yielded a time gain of 15 to 20 seconds per beam for IMRT, depending on the number of segments per beam; and for VMAT a gain of ca. 20 seconds in-between the two arcs. Therefore, if AFS is enabled the delivery times for IMRT reported in this study would be reduced by 1:30 to 3:00 min. It should be noted, however, that our timings neglect possible delays due to interaction with the patient in-between the delivery.

### Data retrieval and statistical analysis

For the purpose of comparison, all participating institutes submitted their completed VMAT and IMRT plans in DICOM RTdose format. The dose distributions were imported into Pinnacle^3^ using an in-house developed import script, if necessary normalized to the minimal required PTV coverage, and DVHs and dose parameters were retrieved for each plan using automated procedures. The data from all five patients and five institutes was pooled by treatment modality and analyzed in SPSS v15.0 as a whole and, in addition, stratified by patient and by institute. Hypothesis testing was performed at 95% confidence level using a paired two-sided Wilcoxon signed-rank test, if not otherwise specified.

## Results

All institutes succeeded in producing clinically acceptable VMAT and IMRT plans for SIB treatments of HNC for all patients. All treatment planners aimed to limit dose to OARs as much as possible and tightened the objectives accordingly as long as the primary goals of the treatment planning protocol were still fulfilled (see Table 2 for a priority listing). Only small differences were found between IMRT plans from different institutes, which may be due to each institute’s preferences and traditions in sparing the different OARs in daily clinical practice. Similar observations were made for the VMAT plans from the different institutes.

**Figure 1:**
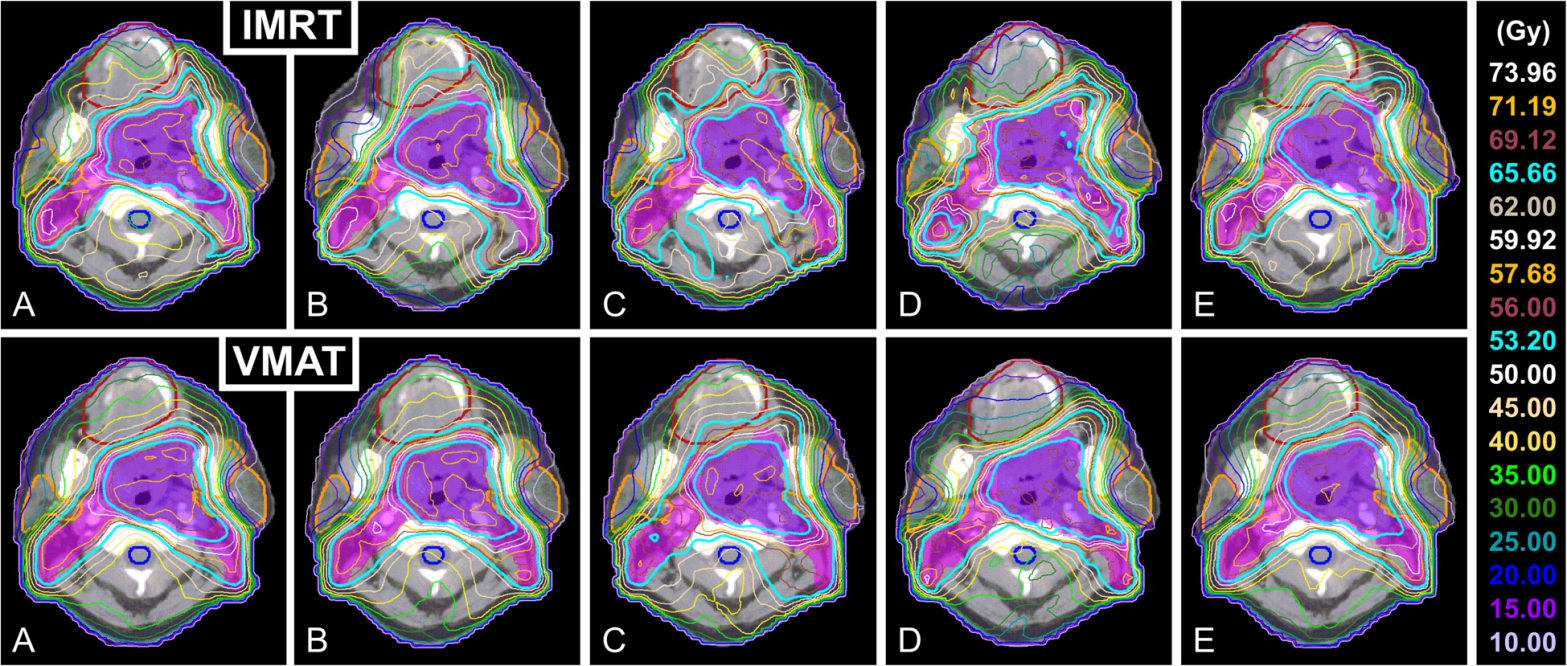
**Dose distributions in a transverse slice for IMRT and VMAT plans of all participating institutes. **Dose distributions in a transverse slice for IMRT and VMAT plans prepared by the participating institutes **A **to **E**. OARs are depicted with a thick solid line: oral cavity (brown), parotid glands (orange) and spinal cord (blue).

**Figure 2:**
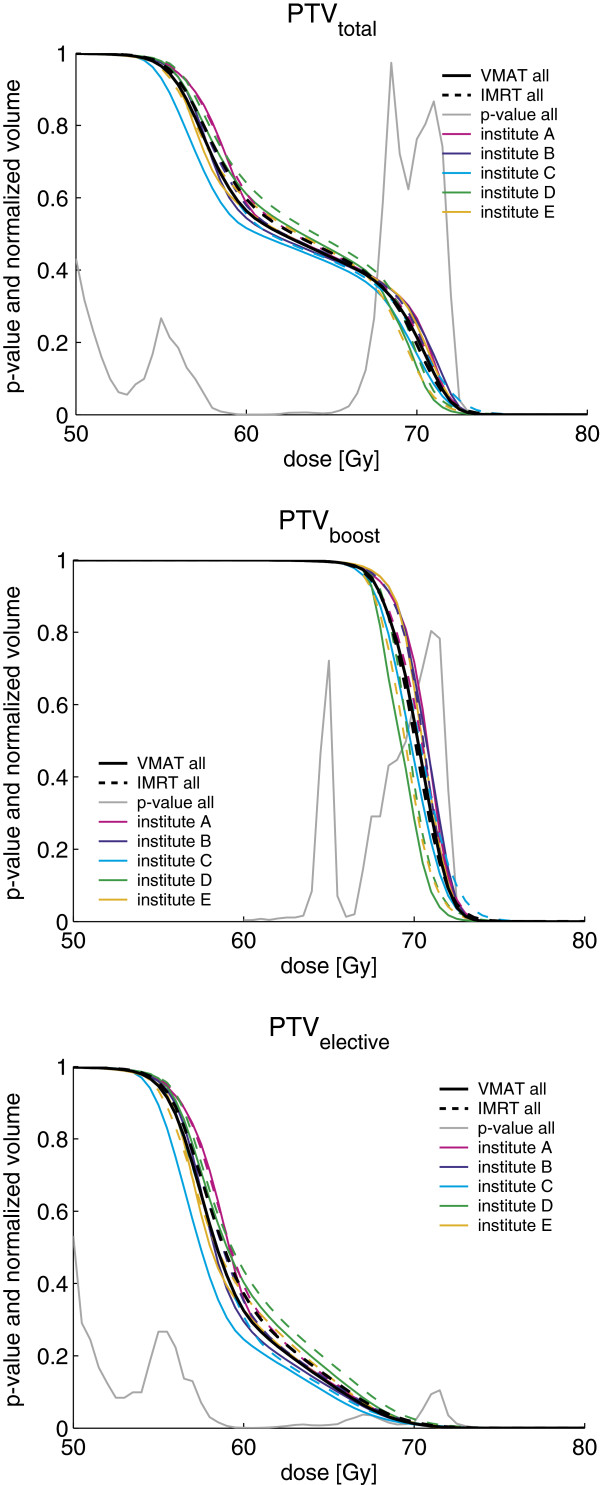
**DVHs for the different PTVs for VMAT and IMRT and p-values for pooled data. **DVHs for PTV_total_, PTV_boost _and PTV_elective _(= PTV_total_ - PTV_boost_) for VMAT (solid line) and IMRT (dashed line). DVHs are shown for pooled data of all institutes (black) and stratified by institute (colors see legend). The p-values shown were obtained for the pooled data using a paired two-sided Wilcoxon signed rank test.

**Figure 3:**
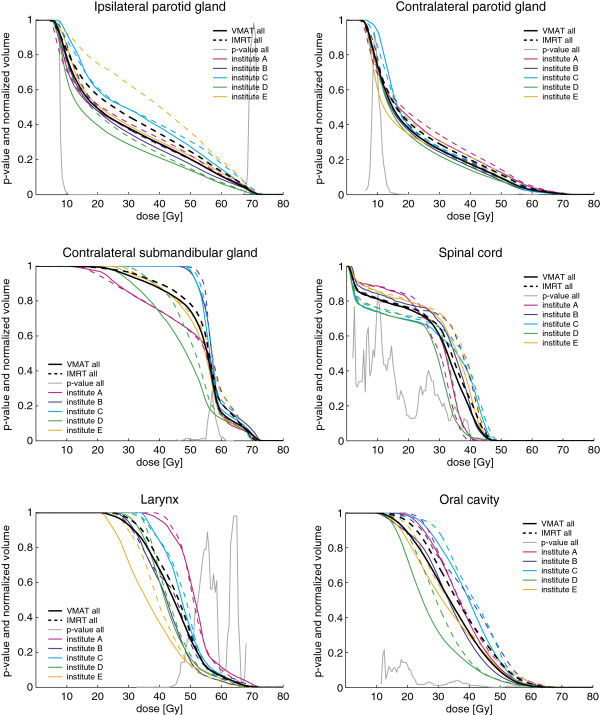
**DVHs for different OARs for VMAT and IMRT and p-value for pooled data. **DVHs for parotid and submandibular glands, spinal cord, larynx and oral cavity for VMAT (solid line) and IMRT (dashed line). DVHs are shown for pooled data of all institutes (black) and stratified by institute (colors see legend). The p-values shown were obtained for the pooled data using a paired two-sided Wilcoxon signed rank test.

Dose distributions of IMRT and VMAT plans for a typical case prepared by the different institutes are shown in Figure 1. Generally, regardless of patient and institute, with VMAT the isodose surfaces encompassed the PTV tighter with similar or better sparing of the OARs. This was reflected in steeper dose fall-offs for the corresponding DVHs of the different PTVs (see Figure 2) and also smaller dose conformity indices (CIs) for VMAT plans. The CI_95_, defined by the ratio of total volume receiving 95% of the prescribed doses (i.e., 53.2 Gy and 65.66 Gy for PTV_elective_ and PTV_boost_, respectively) and the volume of the PTV receiving the same dose, was found to be significantly better with VMAT for both PTV_boost_ and PTV_total_ compared to IMRT for the pooled data (summarized in Table 3); as well when stratified by institute (see Table 4) or by patient (see Table 5). Comparison of the DVHs of different OARs reveals that with VMAT dose to OARs is in almost all cases reduced compared to IMRT (see Figure 3).

**Table 3:** Plan parameters and significance of differences based on the pooled data of five patients and five institutes

		VMAT average ± 1 SD*	IMRT average ± 1 SD*	Average difference ± 1 SD* (VMAT – IMRT)	**p-value (Wilcoxon’s signed-rank test)^**
PTV_boost_	CI_95_	**1.37** ± 0.08	**1.45** ± 0.11	-0.08 ± 0.09	**.001**
PTV_total_	CI_95_	**1.50** ± 0.09	**1.62** ± 0.10	-0.12 ± 0.07	**< .001**
Normal tissue	V_5Gy_ (cm^3^)	*5050* ± 730	*5030* ± 750	-20 ± 260	n.s.
	V_10Gy_ (cm^3^)	*4050* ± 630	*3970* ± 590	-80 ± 160	n.s.
	V_20Gy_ (cm^3^)	2830 ± 510	2860 ± 480	30 ± 150	n.s.
Spinal cord	D_max_ (Gy)	**45.1** ± 3.5	**46.6** ± 3.0	-1.5 ± 2.3	**.001**
	D_1%_ (Gy)	**43.4** ± 3.7	**44.4** ± 3.5	-0.9 ± 2.2	**.005**
	D_mean_ (Gy)	29.3 ± 4.4	29.8 ± 3.6	-0.5 ± 2.4	n.s.
Brain stem	D_max_ (Gy)	46.4 ± 5.4	47.1 ± 4.7	-0.7 ± 4.6	n.s.
	D_1%_ (Gy)	43.8 ± 5.8	44.0 ± 5.5	-0.2 ± 4.9	n.s.
	D_mean_ (Gy)	13.6 ± 3.6	14.5 ± 4.3	-1.0 ± 2.4	n.s.
Parotid gland ipsilateral	V_25Gy_ (%)	**42.9** ± 15.6	**50.3** ± 20.0	-7.4 ± 14.0	**< .001**
	V_39Gy_ (%)	**30.0** ± 14.6	**35.9** ± 18.6	-5.9 ± 11.2	**< .001**
	D_mean_ (Gy)	**28.0** ± 7.5	**31.1** ± 9.1	-3.1 ± 5.1	**< .001**
Parotid gland contralateral	V_25Gy_ (%)	**31.0** ± 7.1	**34.5** ± 6.8	-3.5 ± 3.6	**< .001**
	V_39Gy_ (%)	**18.3** ± 6.0	**20.8** ± 6.1	-2.5 ± 2.5	**< .001**
	D_mean_ (Gy)	**22.0** ± 2.9	**23.3** ± 2.8	-1.3 ± 1.5	**< .001**
Submandibular gland contralateral^$^	V_39Gy_ (%)	88.1 ± 15.3	90.8 ± 13.5	-2.7 ± 6.9	n.s.
	V_60Gy_ (%)	**16.4** ± 21.4	**21.8** ± 27.3	-5.5 ± 8.5	**.020**
	D_mean_ (Gy)	**53.0** ± 5.9	**54.2** ± 6.1	-1.3 ± 1.8	**.027**
Oral cavity	V_25Gy _(%)	**79.8** ± 22.9	**86.3** ± 15.7	-6.5 ± 10.2	**.011**
	V_39Gy _(%)	**40.6** ± 22.0	**48.8** ± 23.3	-8.2 ± 12.4	**.002**
	D_mean _(Gy)	**36.7** ± 7.8	**39.4** ± 7.3	-2.7 ± 2.8	**< .001**
Larynx	V_39Gy_ (%)	**75.4** ± 24.7	**79.1** ± 21.6	-3.8 ± 6.7	**.012**
	V_45Gy _(%)	54.1 ± 27.4	58.2 ± 26.5	-4.1 ± 9.3	n.s.
	D_mean _(Gy)	**45.5** ± 5.3	**46.5** ± 4.4	-1.0 ± 1.5	**.004**
Pharyngeal constrictors°	V_39Gy _(%)	78.7 ± 24.5	81.5 ± 20.0	-2.8 ± 15.2	n.s.
	V_45Gy _(%)	*59.7* ± 29.4	*57.5* ± 25.5	2.2 ± 19.9	n.s.
	D_mean _(Gy)	*47.1* ± 5.3	*46.9* ± 3.8	0.2 ± 2.7	n.s.
Mandible°	V_39Gy _(%)	**74.0** ± 13.1	**78.2** ± 11.8	-4.2 ± 8.4	**.025**
	V_60Gy _(%)	**25.7** ± 15.0	**30.1** ± 16.2	-4.4 ± 5.1	**< .001**
	D_mean _(Gy)	**48.6** ± 5.5	**50.3** ± 5.7	-1.7 ± 1.7	**< .001**
Effective delivery time^#^	(min:sec)	**5:54** ± 1:05	**13:15** ± 1:38	-7:21 ± 1:55	**< .001**
MUs	--	**643** ± 111	**828** ± 149	-185 ± 129	**< .001**

* Standard deviations (SDs) are given as absolute values.

^ Hypothesis testing using a paired t-test results in almost identical significances at 95% confidence level.

^$ ^The contralateral submandibular gland was delineated in only three patients.

° Not all institutes used objectives to control dose for these structures.

^# ^Based on effective delivery times reported by four institutes.

**Table 4:** Plan parameters stratified by institute

		**Institute Technique**	**A**	**B**	**C**	**D**	**E**
PTV_boost_	CI_95_	VMAT	1.39	**1.35**	**1.31**	**1.43**	1.38
		IMRT	1.43	**1.42**	**1.37**	**1.55**	1.47
PTV_total_	CI_95_	VMAT	1.51	**1.49**	**1.54**	**1.45**	**1.51**
		IMRT	1.58	**1.63**	**1.70**	**1.55**	**1.65**
Spinal cord	D_1%_ (Gy)	VMAT	39.1	**45.6**	47.7	41.1	**43.8**
		IMRT	39.7	**46.7**	48.0	42.0	**45.5**
Brain stem	D_1%_ (Gy)	VMAT	*44.1*	45.6	*49.4*	35.4	44.6
		IMRT	*42.9*	45.8	*46.9*	40.0	44.4
Parotid gland ipsilateral	D_mean_ (Gy)	VMAT	**27.6**	**26.6**	**33.3**	**23.2**	**29.3**
		IMRT	**28.5**	**28.4**	**34.2**	**25.3**	**39.0**
Parotid gland contralateral	D_mean_ (Gy)	VMAT	**23.9**	**21.1**	23.4	20.6	**20.8**
		IMRT	**24.8**	**22.7**	23.6	21.5	**23.6**
Submandibular gland contralateral	D_mean_ (Gy)	VMAT	*49.4*	58.0	57.4	47.5	52.6
		IMRT	*49.1*	58.6	58.1	51.1	54.3
Oral cavity	D_mean_ (Gy)	VMAT	40.0	**35.2**	**41.6**	**30.7**	36.0
		IMRT	42.5	**40.2**	**43.6**	**33.5**	37.4
Larynx	D_mean_ (Gy)	VMAT	51.4	**44.5**	*49.4*	43.5	**38.6**
		IMRT	51.5	**46.3**	*49.1*	44.5	**40.9**
Pharyngeal constrictors°	D_mean_ (Gy)	VMAT	***52.0***	45.0	*49.8*	43.9	44.7
		IMRT	***49.0***	45.7	*48.8*	44.2	46.6
Mandible°	D_mean_ (Gy)	VMAT	48.8	48.0	50.5	47.6	**48.0**
		IMRT	49.7	49.9	51.1	49.3	**51.3**
Effective delivery time	(min:sec)	VMAT	**4:51**	**6:50**	**--**	**5:15**	**6:41**
		IMRT	**11:47**	**12:08**	**--**	**15:04**	**14:00**
MUs	--	VMAT	**600**	602	**540**	774	**698**
		IMRT	**830**	670	**824**	841	**975**

Pairs in **bold **denote statistically significant differences between VMAT and IMRT.

Pairs in *italic *denote that the averaged plan parameter is better for IMRT.

° Not all institutes used objectives to control dose for these structures.

**Table 5:** Plan parameters stratified by patient

		**Patient Technique**	**1**	**2**	**3**	**4**	**5**
PTV_boost_	CI_95_	VMAT	**1.37** ± 0.03	1.41 ± 0.06	1.46 ± 0.10	1.30 ± 0.03	1.31 ± 0.03
		IMRT	**1.54** ± 0.09	1.44 ± 0.04	1.50 ± 0.07	1.33 ± 0.05	1.42 ± 0.17
PTV_total_	CI_95_	VMAT	**1.43** ± 0.06	1.58 ± 0.07	**1.52** ± 0.04	**1.57** ± 0.08	**1.40** ± 0.04
		IMRT	**1.53** ± 0.04	1.71 ± 0.09	**1.63** ± 0.06	**1.67** ± 0.10	**1.57** ± 0.11
Spinal cord	D_1%_ (Gy)	VMAT	**42.1** ± 5.5	44.7 ± 2.6	43.4 ± 3.5	44.0 ± 4.1	42.9 ± 3.6
		IMRT	**43.8** ± 4.3	44.3 ± 2.9	44.8 ± 2.4	44.5 ± 4.7	44.4 ± 4.2
Brain stem	D_1%_ (Gy)	VMAT	48.3 ± 4.7	**42.1** ± 6.0	45.5 ± 5.6	*41.4* ± 6.9	***41.7*** ± 5.0
		IMRT	48.2 ± 1.9	**45.7** ± 4.3	47.6 ± 4.2	*40.5* ± 3.6	***37.9*** ± 5.0
Parotid gland ipsilateral	D_mean_ (Gy)	VMAT	**21.7** ± 2.0	**30.3** ± 4.9	**24.8** ± 3.5	**38.1** ± 9.2	**25.1** ± 2.4
		IMRT	**23.6** ± 1.7	**33.3** ± 3.5	**30.5** ± 10.2	**41.8** ± 11.0	**26.3** ± 3.2
Parotid gland contralateral	D_mean_ (Gy)	VMAT	**21.1** ± 1.9	**23.1** ± 1.0	22.1 ± 2.1	25.0 ± 2.9	**18.6** ± 1.9
		IMRT	**23.2** ± 1.9	**24.1** ± 1.3	24.2 ± 0.5	25.3 ± 3.5	**19.4** ± 1.5
Submandibular gland contralateral	D_mean_ (Gy)	VMAT	49.2 ± 6.0	**55.0** ± 6.3	--	--	54.8 ± 4.3
		IMRT	49.6 ± 5.6	**57.4** ± 6.4	--	--	55.7 ± 3.7
Oral cavity	D_mean_ (Gy)	VMAT	**31.3** ± 4.8	34.7 ± 5.4	**33.3** ± 7.0	47.7 ± 1.7	36.5 ± 7.4
		IMRT	**34.2** ± 4.8	38.2 ± 3.1	**37.4** ± 8.9	48.8 ± 1.7	38.7 ± 7.2
Larynx	D_mean_ (Gy)	VMAT	43.2 ± 4.4	44.9 ± 7.3	47.1 ± 4.6	46.8 ± 5.5	45.3 ± 5.5
		IMRT	44.5 ± 4.3	45.5 ± 5.8	47.5 ± 4.2	47.7 ± 4.7	47.0 ± 3.9
Pharyngeal constrictors°	D_mean_ (Gy)	VMAT	*42.3* ± 3.9	*50.5* ± 6.6	48.9 ± 2.6	*48.0* ± 4.9	45.7 ± 5.2
		IMRT	*42.1* ± 2.7	*48.2* ± 3.7	49.3 ± 2.3	*47.9* ± 3.6	46.9 ± 2.6
Mandible°	D_mean_ (Gy)	VMAT	41.7 ± 1.3	45.1 ± 1.4	55.2 ± 1.7	54.2 ± 1.5	**46.7** ± 1.7
		IMRT	42.4 ± 0.9	47.2 ± 1.2	57.6 ± 1.5	55.2 ± 1.6	**48.9** ± 1.7
Effective delivery time	(min:sec)	VMAT	5:38 ± 0:42	6:00 ± 0:48	6:22 ± 1:28	6:07 ± 1:31	5:26 ± 1:04
		IMRT	12:52 ± 0:41	13:09 ± 2:18	13:46 ± 1:07	13:30 ± 1:50	12:57 ± 2:23
MUs	--	VMAT	**581** ± 55	641 ± 90	735 ± 170	**668** ± 91	**589** ± 73
		IMRT	**702** ± 92	836 ± 166	975 ± 125	**855** ± 100	**773** ± 142

Pairs in **bold **denote statistically significant differences between VMAT and IMRT.

Pairs in *italic *denote that the averaged plan parameter is better for IMRT.

° Not all institutes used objectives to control dose for these structures.

**Figure 4:**
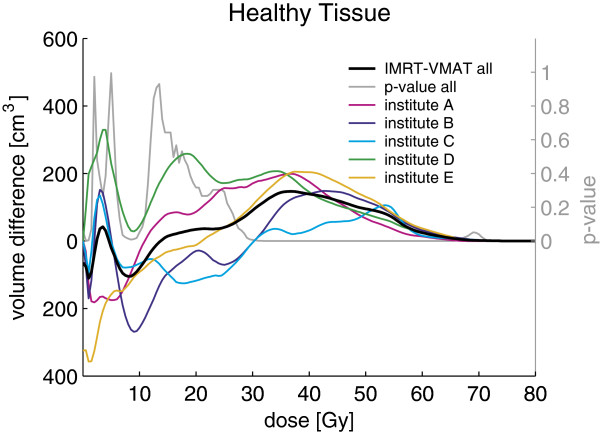
**Averaged absolute volume difference between DVHs for healthy tissue and p-value for pooled data. **Averaged absolute volume difference between DVHs for IMRT and VMAT for healthy tissue for the pooled data (black) and stratified by institute (colors see legend). The p-values shown were obtained for the pooled data using a paired two-sided Wilcoxon signed rank test.

In addition, with VMAT the isodose surfaces encompassed the PTVs more smoothly (i.e. less occurrence of high dose bulges reaching far outside the PTVs) and fewer hot spots outside the PTVs were observed (see Figure 1 for a typical example). This was also reflected in reduced volumes of healthy tissue receiving doses above 30 Gy for VMAT compared to IMRT. This is illustrated by the average absolute volume difference between both techniques shown in Figure 4.

### VMAT vs. IMRT based on pooled data

Comparison of the plan parameters for VMAT and IMRT plans generated for the same patient by the same institute showed significantly better sparing for most OARs (see Table 3). Figure 4 shows averaged DVHs for the pooled data for different OARs for VMAT (black solid line) and IMRT (black dashed line) and corresponding p-values (grey solid line). The average maximum point dose and D_1%_ (1% of the volume receives more than this dose) to spinal cord and brain stem were lower with VMAT, though only for the spinal cord at significant level. With VMAT we observed a significantly lower average mean dose and V_39Gy_ (i.e. the percentage of volume receiving more than 39 Gy) for ipsilateral and contralateral parotid glands, mandible, oral cavity and larynx. In addition, the V_25 Gy_ was significantly improved for the parotid glands and the oral cavity. The contralateral submandibular gland was delineated in only three out of the five patients and was always located in very close proximity to the PTV. For this reason, we generally observed high doses to the contralateral submandibular gland with both VMAT and IMRT (see DVHs in Figure 3) and the plan acceptance parameters could not be fulfilled in any case.

VMAT delivery times estimated by the TPSs for both arcs in the dual arc were on average 3:56 min and ranged from 3:00 to 5:43 min, depending on each institute’s practice and difficulty of the case. In almost all cases, the TPS made use of the maximum delivery time allocated by the planner. The effective delivery times for VMAT (defined as the time from start of the first arc and end of the second arc) based on reports from four institutes were on average 5:54 min and ranged from 4:18 to 7:57 min. One institute could not report effective delivery times for their VMAT plans due to a delay with installing VMAT licenses for their treatment machines. The delivery times of the IMRT plans (defined from start of the first segment of the first beam till end of the last segment of the last beam) were on average 13:15 min and ranged from 9:40 to 15:56 min. Based on these results, with VMAT for HNC the delivery times can be expected to be reduced by ~50% compared to IMRT.

### Evaluation per institute

When stratifying the data by institute the overall picture regarding improved plan parameters with VMAT remained the same. For all plan parameters listed in Table 4 with exception of the contralateral submandibular gland, larynx and brain stem VMAT yielded better results, regardless of institute, though not always at statistically significant level. Please note that statistical power of the stratified comparison is low since the statistical analysis is based on only five pairs. However, the mean dose to the ipsilateral parotid gland was in all cases significantly better for VMAT, and for most institutes this also held for the contralateral parotid gland.

The data listed in Table 4 also provides interesting information on each institute’s planning preferences. For institute C dose conformity to the PTV_boost_ had probably a relative high priority compared to other institutes, and sparing of the ipsilateral parotid gland had lower priority, as well as achieving low maximal doses to spinal cord and brain stem. Institute A seemed to give high preference to an as low as possible maximal point dose in spinal cord and brain stem, whereas institutes B, D and E probably tried to achieve a as low as possible mean dose to the contralateral parotid gland, and institute D even tried this for the ipsilateral parotid gland. Probably, institute D profitted here from using a beam modulator that supports interdigitation of the MLC leaves, which allows a higher degree of dose modulation.

Institute D also seemed to pay increased attention to low doses to the oral cavity while dose conformity to the PTVs seemed to play a less important role. These findings were also supported by the underlying dosimetric data of the individual patients, where almost all respective pairings of dosimetric parameters showed the same preferences as the averages per institute listed in Table 4. This supports our idea that even within quite defined boundary conditions for treatment planning, including a beforehand agreed treatment protocol and guidelines for sparing OARs, quite some variation is possible in the results of treatment planning studies like the one presented here.

### Evaluation per patient

Likewise, stratification of the data by patient does not largely change the previous findings. All plan parameters except the mean doses to the larynx were lower for VMAT compared to IMRT, though not all at significant level (see Table 5). A reason for this observation could be that the larynx is located in-between the two sides of the PTV_elective_. With VMAT dose is delivered from many directions and therefore more smeared out on OARs in such constellations; whereas with IMRT large parts of the dose are often given from directions omitting the OARs in-between the PTV.

## Discussion

Analysis of the data resulting from this multi-institutional collaboration on the clinical introduction of VMAT for HNC shows that VMAT plans including a double arc generated with Pinnacle^3^ or Oncentra Masterplan have an improved plan quality compared to IMRT plans with 5–9 beam ports. This observation is valid independently of institution-specific planning strategies, choice of the set of objectives, or preferences for sparing OARs. Based on the effective delivery times reported from four institutes we expect a reduction in the effective delivery times of ~50% with VMAT compared to IMRT. The decreased treatment delivery time obtained with VMAT will improve patient comfort and result in a smaller impact of intra-fraction movements, as described by Hoogeman *et al*. [17].

Several single-institution studies comparing VMAT and IMRT for HNC have been reported in literature, most based on RapidArc [9,10] and on SmartArc [11-15]. The strength of the study presented here bases on its multi-institutional setup and the rather limited regulations regarding the planning environment and procedures, resulting in varying solutions for the same set of patients. All plans were generated using planning environment and procedures that were (or will shortly be) adopted for routine clinical use. As such, the data presented in this study reflects a broad range of clinically achievable and acceptable results and, therefore, allows a planner-independent evaluation of the potential of VMAT vs. IMRT for irradiation of HNC.

IMRT has been the standard for radiation therapy of advanced HNC in all participating institutes for several years by now, and consequently, all institutes had a similar level of experience in IMRT treatment planning when starting this study. All participating institutes performed IMRT and VMAT treatment planning using the same CT data sets including contouring, ensuring excellent comparability of the data between institutes. The TPSs employed in this study, Pinnacle^3^ and Oncentra, both make use of nearly identical VMAT and IMRT optimization modules developed by RaySearch Laboratories. Therefore, the data retrieved from these two TPSs was pooled and analyzed as one population.

Pair-wise analysis of VMAT and IMRT plans within the same patient and institute showed for almost all plan pairs better sparing of OARs and dose conformity with VMAT (please note that statistical power is low in the stratified comparison). A major reason for this could be the rotational character of VMAT which allows dose delivery from many more directions than with static-beam IMRT with mostly 5–9 beam ports used. With VMAT optimization dose is automatically redistributed along the arc, which means that to a certain extent beam angle optimization is inherent to VMAT. The additional degrees of freedom with VMAT lead to the better dose conformity, which in turn allows for a better sparing of OARs in close proximity to the PTVs.

Further exploration of the underlying dosimetric data yielded that one institute chose to completely sacrifice one of the parotid glands with IMRT for some patients, but was able to spare it quite successfully with VMAT. Already in 1996, Eisbruch *et al*. reported on a 3D-CRT technique to spare the contralateral parotid gland, while deliberately accepting underdosage in the surrounding target volume with supposedly “lower” risk to contain disease [18]. With the introduction of IMRT, this technique was refined and the contralateral gland could be spared without hazarding underdosage in the target volume; however, often at the price of still sacrificing the ipsilateral gland [19]. It seems that the paradigm “*sacrificing one parotid gland to achieve better sparing of the contralateral gland*” often applied with advanced HNC can be revisited with the advent of VMAT.

From Table 4 we can see that some institutes have only a few dosimetric parameters with significant statistical difference, whereas others have the majority of parameters showing significant differences between VMAT and IMRT. A reason for this may be that only limited efforts were made to homogenize the IMRT techniques among institutes before starting the study. The dosimetric gain of VMAT compared to each of the individual IMRT techniques may therefore differ depending on how complex/well-designed an IMRT technique of a specific institute was compared to those of other institutes. Keeping these “heterogeneous” sources in mind, the conclusions of the presented study with VMAT leading to better dosimetric results are even more striking.

Limitations of the here presented study include the small sample size in the stratified comparisons, heterogeneity in TPS and equipment of the participating institutes, and limitation to a specific IMRT and VMAT implementation combined with specific linear accelerator equipment. For the comparisons stratified by institutes or patients the sample size of only five institutes and five patients is small, resulting in a low statistical power in the stratified comparisons of VMAT and IMRT. However, the pooled data with *n = 25* allows drawing firm conclusions since for each pairing we can assume statistical independency as different treatment planners are involved. Regarding the heterogeneity of equipment, the two different TPSs employed in this study may yield slightly different results for both IMRT and VMAT plans, although the optimization modules in both TPSs are of the same evolutionary origin. Regarding the different MLCs used for treatment planning, the newer generation beam modulator with narrower leaves of 4 mm width also allowing interdigitation may lead to better dosimetric results for both IMRT and VMAT, especially regarding dose conformity, due to increased degrees of freedom in leave motion. However, any differences in boundary conditions will be balanced since only pairs of IMRT and VMAT plans achieved under the same conditions are compared. The superior results for VMAT obtained in this study may be valid only for the specific combination of step-and-shoot IMRT and SmartArc/Oncentra VMAT delivered on Elekta linear accelerators equipped with the specified MLCs, and a similar comparison with sliding-window IMRT could lead to different results.

The data presented in this planning study comparing VMAT and static-beam IMRT for HNC resulted from a collaboration of five institutes in the Netherlands with similar equipment, aiming at a safe and fast clinical introduction of VMAT for HNC. Discussion of the planning results and exchange of ideas and information regarding VMAT treatment planning parameters and objectives between participating institutes during the collaboration resulted in noticeable improvement of the VMAT plans. We would like to stress that collaboration between institutes with similar equipment and treatment planning software on the clinical introduction of a new treatment modality can help to efficiently steepen the learning curve and to achieve a high quality of treatment planning within a short time. Problems and questions arising can be solved during the discussions within the group, and as a result all institutes require less time and effort for the clinical introduction of the advanced treatment modality VMAT for HNC.

## Conclusions

VMAT plans including double arcs for simultaneous-integrated boost treatments of head-and-neck cancer were found to be improved compared to static-beam step-and-shoot IMRT plans including 5–9 beam ports regarding dose to OARs and dose conformity, while delivery times were significantly shortened by 50%.

## Competing interests

The authors declare that they have no competing interests.

## Authors’ contributions

AH participated in the study design and study coordination, performed treatment planning, collected and analyzed data, performed the statistical analyses for the study, interpreted data, revised literature and drafted the manuscript. DvG participated in the study design, prepared patient data sets for treatment planning, interpreted data, revised literature and helped draft the manuscript. MA, EK, DS, MK and RL participated in the study design, performed treatment planning, interpreted data, revised literature and helped draft the manuscript. CvV participated in the study design and study coordination, interpreted data, revised literature and helped draft the manuscript. All authors read and approved the final manuscript.
